# Supplementary material for: Straw application promotes soil carbon storage by affecting aggregate-associated bacterial community structure and RuBisCO activity: a 35-year field experiment
Source: Microbiol Spectr. 2025 Jul 22;13(9):e00088-25. doi: 10.1128/spectrum.00088-25 (PMC12403595; doi:10.1128/spectrum.00088-25)
Supplement: Supplemental material — Table S1; Fig. S1 [file spectrum.00088-25-s0001.docx]

**Supplementary material**

Table S1 PERMANOVA analysis of treatment effects on bacterial community structure.

| Characteristics | SumsOfSqs | MeanSqs | F.Model | R^2^ | P.value |
| --- | --- | --- | --- | --- | --- |
| Aggregate size | 0.071 | 0.024 | 2.369 | 0.182 | 0.049 |
| Measure | 0.044 | 0.024 | 2.075 | 0.112 | 0.113 |
| M×A | 0.216 | 0.020 | 2.701 | 0.553 | 0.004 |


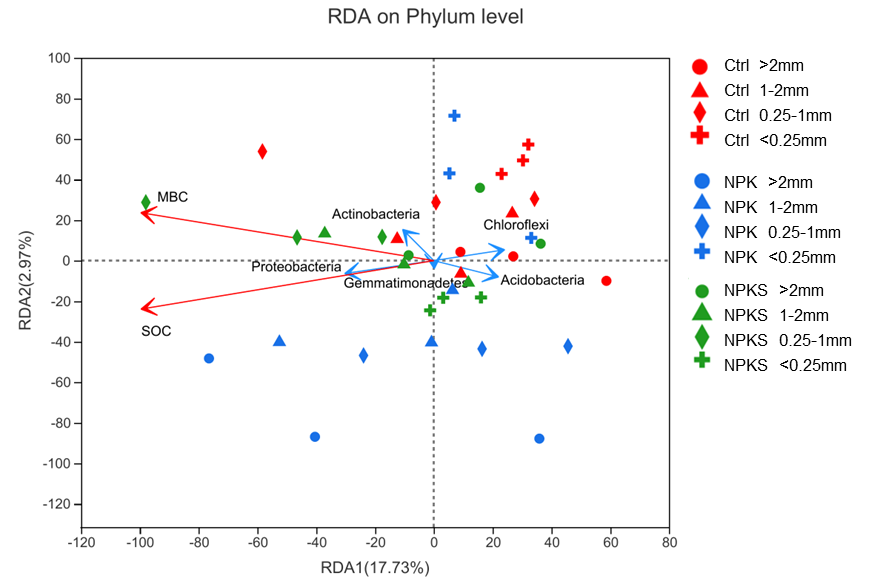


Fig.S1 Redundancy analysis (RDA) showed the relationship between SOC, MBC and bacterial community (phylum level). The top five dominant bacterial phyla are shown.
